# Contribution of Green Propolis to the Antioxidant, Physical, and Sensory Properties of Fruity Jelly Candies Made with Sugars or Fructans

**DOI:** 10.3390/foods10112586

**Published:** 2021-10-26

**Authors:** Cristina Cedeño-Pinos, María Cristina Marcucci, Sancho Bañón

**Affiliations:** 1 Department of Food Technology and Science and Nutrition, Veterinary Faculty, Regional Campus of International Excellence “Campus Mare Nostrum”, University of Murcia, 30100 Murcia, Spain; cristinacarmen.cedenop@um.es; 2 Departament of Biosciences and Oral Diagnosis, Instituto de Ciência e Tecnologia, Universidade Estadual Paulista ICT-Unesp, Campus de São José dos Campos 12245-000, Sâo Paulo 01000-000, Brazil; cristina.marcucci@unesp.br

**Keywords:** propolis, polyphenols, Artepillin-C, antioxidants, functional candy

## Abstract

Enrichment with phenolic compounds is proposed as a strategy to obtain more stable and healthier candy products. A green propolis ethanolic dry extract (PEE) from *Braccharis dracunculifolia* (Brazilian Alecrim-do Campo) was assessed as an antioxidant in jelly candies. Three levels (0, 0.01, and 0.02% *w*/*w*) of PEE were tested in jelly candies alternatively made with two carbohydrate bases (sugars or fructans) and three fruity dyes and flavours (menthe, orange, or strawberry). Propolis polyphenol content (identified by HPLC-MS and quantified by HPLC-DAD/UV-Vis), antioxidant capacity (total phenolics and radical scavenging activity), physical properties (moisture, pH, CIELab colour, and texture profile analysis), and flavour were studied in candies. PEE was rich in polyphenols (>8.7%), including several prenylated p-coumaric, caffeoylquinic and diterpenic acids, and flavonoids, with Artepillin-C (3.4%) as the main bioactive compound. The incorporation of PEE into the hot liquor at 80 °C for 5 min before moulding allowed a good retention of propolis polyphenols in the final product (recovery percentages of up to 97.4% for Artepillin-C). Jelly candies made with sugars or dietetic fructans have poor antioxidant properties, which depend on the dyes and flavours used. Using PEE (at 0.02%) strongly improved the antioxidant capacity (relative increases of up to 465%) of candies without altering the pH, colour, or texture, although off-flavour may appear. Propolis, due to its good antioxidant properties, has potential for use as a functional ingredient in jelly candies.

## 1. Introduction

Jelly candies are popular products based on sugars and gelatine with a wide margin for nutritional improvement. There is currently a growing reluctance to consume food rich in calories and sugar owing to their negative repercussions on health (obesity, diabetes, etc.); therefore, dietetic jelly candies made with healthier ingredients are being developed to satisfy this demand. Among the possible strategies, the use of fructan fibres (inulin and fructooligosaccharides, FOS) combined with natural sweeteners (polyalcohols and *Stevia rebaudiana*) has been shown to have good properties for producing less-caloric jelly candies [1,2]. This strategy can be complemented using natural antioxidants. Vitamin C is often used in candy products, including jelly candies, although ascorbic acid is prone to degradation during cooking and further storage [3]. In the last years, several phenolic ingredients, including fruit derivatives, such as raspberry and orange powder [4], pomegranate juice and apple puree [5], açai concentrate [6], soursop fruit [7], and strawberry guava pulp [8], as well as plant extracts, such as menthe and camomile [9], white tea [10], lycium and hovenia [11], ginger [12], kecombrang flower [13], soursop leaves [7], and guajava leaf [14], have been tested for developing functional jelly candies. In general, such phenolic ingredients enhanced the antioxidant capacity (AC) of candies, although some, particularly, plant extracts, may provide some unpleasant sensory properties (bitterness, astringency, herbal off-flavours, etc.), which limits their possible use in widely consumed products. A previous study [2] evidenced the good chances of using micro quantities (up to 0.026% *w*/*w*) of rosemary extract, a product with an intense off-flavour, as an antioxidant in fruity jelly candies, without any sensory detriment and with a good retention of polyphenols. Thus, other phenolic extracts might also be used to obtain more stable and healthier candies.

Propolis is a natural product collected by honeybees in plant exudates mixed with wax and other own substances. It is considered a natural source of polyphenols, including different phenolic acids and flavonoids, that may vary largely depending on the raw material collected by the bees [15]. Propolis has been reported to have various health benefits related to gastrointestinal disorders, allergies, and gynaecological, oral, and dermatological problems [16]. Different types of propolis are known worldwide: green Brazilian propolis (having *Baccharis dracunculifolia* as the major plant source), red Brazilian propolis (from *Dalbergia ecastaphyllum* and *Symphonia globulifera*), European and Chinese propolis (*Populus* spp.), Russian propolis (*Betula verrucosa Ehrh*), and Cuban and Venezuelan red propolis (*Clusia* spp.), among others [15,17,18]. Some types of propolis have high market value for their medicinal properties, such as Brazilian green propolis from *Baccharis dracunculifolia* [19]. Alcoholic extracts of this green propolis are particularly rich in non-volatile polyphenols, including phenolic acids (e.g., p-coumaric, 3,5-diprenyl-4-hydroxycinnamic or Artepillin-C, and caffeoylquinic) and flavonoids (e.g., kaempferol and kaempferide) [20].

Propolis use as a food supplement is authorized by the European Food Safety Agency (EFSA) [21] and the National Health Surveillance Agency in Brazil [22]. An oral dose of 200 mg/day/person has been recommended for propolis used as a health food or dietary supplement [23]. Daily intake of propolis has no recommended dosage as yet, although it is presumed that one ranging from 260 to 2870 mg/day/person would be safe in humans, and that, in the case of Brazilian green propolis, nutraceutical dosages would be around 500 mg/day/person [24]. Propolis can be used as a nutraceutical supplement in the form of liquids, effervescent, tablets, and pills, alone or in combination with other natural products [25]. At present, propolis is also being assessed by the EFSA as a natural preservative for food application [26]. Propolis extracts have been demonstrated to be effective as antimicrobial [27], antioxidant [28], and edible egg coating [29] in food products. Hard and jelly candies with honey and propolis are being commercialized, although available research is scarce. It was reported that functional jelly candies containing high levels of propolis show excellent antioxidant properties before and upon in vitro digestion compared to commercial jelly candies [4,30]. Thus, propolis could also be used to improve the oxidative stability and nutritional value of widely consumed jelly candies. However, propolis extracts have been seen to exhibit certain sensory limitations as a food ingredient due to their intense flavour [31]. Therefore, a technological assessment is required for propolis to be properly incorporated into jelly candy formulations. Different aspects, including the retention of bioactive compounds and the resulting antioxidant, sensory, and physical implications should be elucidated. Jelly candies are acidic products of low activity water, whose microbial safety can be ensured by applying mild temperatures [32], which may facilitate the retention of propolis bioactive compounds. Moreover, adverse sensory effects of propolis may be masked with flavours, acids, and dyes. Whatever the case, the resulting effects of propolis polyphenols might change when interacting with different candy ingredients (carbohydrates, dyes, and flavours, etc.) under different cooking conditions. The objective of the present study was the enrichment with propolis antioxidants of fruity jelly candies in order to obtain more stable and healthier products. A green propolis ethanolic dry extract (PEE) from *Braccharis dracunculifolia* was assessed as an antioxidant ingredient in jelly candies based on sugars or fructan fibres. Propolis polyphenol content, antioxidant capacity (total phenolics and radical scavenging activity), physical stability (moisture, pH, colour, and texture profile), and flavour were investigated in candies.

## 2. Materials and Methods

### 2.1. Experimental Design

Jelly candies made with PEE were used in the experiment. A randomized statistical design was performed with three treatments: (i) PEE dose (% *w*/*w*): 0 (untreated), 0.01 (P1), and 0.02 (P2); (ii) carbohydrates: sugars plus starch (S) and fructans plus stevia (F); and (iii) fruity type: menthe, orange, and strawberry. Thirty-six manufacturing batches were sampled (3 PEE levels × 2 carbohydrate basis × 3 fruity types × 2 replicates). Propolis polyphenols were determined in both PEE and jelly candies. Antioxidant capacity, physical properties (moisture, pH, colour, and texture), and flavour were assessed in jelly candies. A one-way variance analysis was used to determine the effects of treatments on the dependent variables, using the Tukey test (*p <* 0.05) for mean comparisons. A number from 18 (physical-chemical variables) to 140 (sensory variables) candy units per treatment and level were sampled. Correlations were calculated as Pearson’s coefficients (*p <* 0.05). The software used was Statistix 8 package for Windows 8.0 (Analytical Software, Tallahassee, FL, USA).

### 2.2. Propolis Ethanolic Extract

A standardized dry PEE was provided by the Apis Brasil company (Pindamonhangaba, São Paulo, Brazil). Fresh green propolis from *Braccharis dracunculifolia* was collected on 25 January 2019. For PEE obtention, 30 g of propolis were triturated with 100 mL of ethanol and kept under magnetic stirring at 40 °C for 24 h. The supernatant was then filtered and dried under vacuum in rotary evaporator. Dry PEE was stored at room temperature until further use. The supplier’s declaration for the product was: phenolic total compounds (9.92 g Gallic Acid Equivalent per each 100 g) determined with the Folin–Ciocalteu method [33]; and wax impurities (9.22 g/100 g) determined with the Soxhlet method [34].

### 2.3. Jelly Candy Manufacturing

Jelly candies were manufactured in a food technology pilot plant located in the University of Murcia, Spain. Candy ingredients and their proportions are indicated in Table 1. The S-candies were made with sucrose (Zukán, Molina de Segura, Murcia, Spain), glucose syrup 79 °Brix (°B) (Zukán), and acid-thinned corn starch (Cleargum Mb 76, Roquette Laisa, Valencia, Spain). The F-candies were made with FOS (Fosvitae chemical synthesis 72 °B, Zukán), Orafti GR chicory inulin (Beneo, Tienen, Belgium), and *Stevia rebaudiana* powder (Zukán). The common ingredients were: type A pork gelatine (Juncà Gelatines, Barcelona, Spain), citric acid (Helm Iberica, Alcobendas, Madrid, Spain), lactic acid (Brenntag Chemistry, Dos Hermanas, Seville, Spain), sodium citrate (Azelis, Cornellá de Llobregat, Barcelona, Spain), dye E120 carminic acid (Bright’nRED Carmine 50 WS powder, Vitiva, Marcovic, Slovenia), yellow dye curcumin E100 (Capcolor yellow 100 WSS, CHR Hansen, Daganzo de Arriba, Madrid, Spain), blue dye E133 idacol (Brilliant blue FDF, Roha Europe, Torrent, Valencia, Spain), strawberry flavour (PIM, Scentium Flavours, Alhama de Murcia, Murcia, Spain), Navelate NF 227626A orange flavour (IGH Flavours & Technology, Alcantarilla, Murcia, Spain), and menthe flavour (IGH Flavours & Technology). Suppliers did not state the composition of fruity flavours.

The pork gelatine was previously dissolved in hot (80 °C) water (2:1 *w*/*w*) while stirring for 30 min. The cooking procedure was adapted to candy ingredients. For S-candies, sucrose, corn glucose syrup, and starch water dispersion were homogenized by stirring and heating at 120 °C for 5 min. After cooking, the temperature of the product (hot liquor) was reduced to 80 °C and the gelatine water solution added and homogenized for 10 min. For F-candies, inulin was mixed with the FOS solution and homogenized with an Ultraturrax (887 g) at room temperature for 5 min until a cream was obtained. The inulin cream and gelatine solution were mixed at 80 °C under stirring for 10 min. The respective water solutions (previously stirred for 10 min at 25 °C) containing acids, flavours, and dyes were then transferred and the liquor was homogenized for 5 min at 80 °C. Optionally, dry PEE was added or not along with the above solutions. Before depositing, the total soluble solids of hot liquor were adjusted to 78 ± 0.1 °B using a hand refractometer (Atago Co., Ltd. Minato-ku, Tokyo, Japan) and was then poured into the starch powder moulds (printed in trays), which were previously conditioned at 30 °C and 10% relative humidity (RH) for 24 h. The trays containing hot jellies were kept in a cooling-drying chamber with air circulation at 21 °C and 35% RH for 24 h. After drying, the jellies were demoulded, waxed with carnauba wax, and kept in polypropylene bags in darkness at 25 °C for 7 days until analysis. The final average weight of jelly candy units was 2.2 ± 0.2 g. Candy images are available in a previous study [32].

### 2.4. Sample Extraction for Polyphenol Quantification and Antioxidant Assays

First, 2 g of sample were melted at 60 °C and dissolved in 10 mL of methanol (Fisher Scientific, Madrid, Spain) using a calibrate flask. Sample solution was stirred for 10 min at 25 °C and then centrifuged at 2580× *g* for 10 min (D2010, Kubota, Tokyo, Japan). The resulting supernatant was collected and centrifuged again in an Eppendorf tube at 6596× *g* for 10 min (D-37520 Biofuge Pico centrifuge, Heraeus, Germany). The supernatant was stored at −20 °C for later use in assays.

### 2.5. HPLC Analysis

The separation and analysis of dry PEE samples were performed with a HPLC/MS system consisting of an Agilent 1290 Infinity II Series HPLC (Agilent Technologies, Santa Clara, CA, USA) equipped with an Automated Multisampler module, a High Speed Binary Pump, and a DAD (Diodes Array Detector) module, and connected to an Agilent 6550 Q-TOF Mass Spectrometer (Agilent Technologies, Santa Clara, CA, USA) using an Agilent Jet Stream Dual electrospray (AJS-Dual ESI) interface. Experimental parameters for HPLC and Q-TOF were set in MassHunter Workstation Data Acquisition software (Agilent Technologies, Rev. B.08.00). Samples (20 µL) were thermostatted at 5 °C and injected onto a Teknokroma Brisa LC2 C18 (4.6 × 150 mm, 5 um) HPLC column, at a flow rate of 0.8 mL/min. The column was equilibrated at 25 °C. Solvents A (MilliQ water with 0.1% formic acid) and B (methanol) were used for the compound separation. After the injection, compounds were eluted using the following gradient (min/% methanol): 0–29 min/30%; 30–36 min/80%; 37–43 min/90%; and 44–50 min/30%. Absorbance signals at 280 and 340 nm were recorded. The mass spectrometer was operated in the negative mode. The nebulizer gas pressure was set to 50 psi, whereas the drying gas flow was set to 16 l/min at a temperature of 200 °C, and the sheath gas flow was set to 12 L/min at a temperature of 300 °C. The capillary spray, nozzle, fragmentor, and octopole 1 RF Vpp voltages were 4000, 200, 350, and 750 V, respectively. Centroid data in the 100–1100 *m*/*z* range were acquired for MS scans in 2 GHz Extended Dynamic Range High Resolution mode with 4 spectra/s, 250 ms/spectrum, and 2026 transients/spectrum. Reference masses at 112.985587 and 1033.988109 *m*/*z* were used for mass correction during the analysis. Data analyses were performed with MassHunter Qualitative Analysis Navigator software (Agilent Technologies, Rev. B.08.00).

Quantification of the polyphenols in PEE and candy samples was performed using standards with the same UV spectrum [35]. P-coumaric acid, prenylated p-coumaric acids, such as 3,5-diprenyl-4-hydroxy cinnamic acid (DHCA), and cinnamic acid derivatives were quantified using p-coumaric (P-CUM) acid (Pubchem CID 1549106) (Sigma-Aldrich Chemistry, Tres Cantos, Madrid, Spain), while caffeic and caffeoylquinic acids were quantified using caffeic acid (Pubchem CID 689043) (Sigma-Aldrich). Stock solutions of caffeic acid (1000 μg/mL) and P-CUM acid (1000 μg/mL) were diluted in 10 mL of methanol using calibrated flasks (10, 15, 20.... 50 μg/mL). The limits of detection (LOD) and quantification (LOQ) were determined using the average standard deviation of response (SD) and the slope of calibration curve (S) according to the formulas LOD = 3.3 (SD/S), LOQ = 10 (SD/S). Standards for the quantification of 2,2-dimethyl-2H-1-benzopyran-6-propenoic acid and Kaempferide were not available. The recovery percentage (%) of P-CUM and DHCA in candies was calculated as 100× g final/added compound on a dry basis.

### 2.6. Total Phenolic Content (TPC)

The TPC was determined using a modified version of the Folin–Ciolcateu method [33]. First, 5 mL of distilled water, 250 µL of sample extract, and 800 µL of Folin–Ciocalteu reagent were transferred to a 10-mL volumetric flask under stirring. After 8 min, 1.2 mL of sodium carbonate water solution (1:5 *w*/*v*) were added to the sample mixture, which was completed with distilled water and kept in a water bath at 20 °C for 2 h. Sample absorbance was measured at the 760 nm wavelength using a UV-VIS spectrophotometer (Unicam, Cambridge, United Kingdom). Gallic acid (PubChem CID 370) was used as a standard (Merck KGaA, Darmstadt, Germany). A calibration line (R^2^ = 0.9992) in concentrations from 0.1 to 5.5 µg GA/mL was used for quantification. Results were expressed as mg Gallic Acid Equivalent (GAE) per 100 g candy.

### 2.7. 2.2’-. Azinobis-3-ethylbenzothiazoline-6-sulfonic (ABTS^•^) Radical Cation Decolouration Assay

The ABTS assay was performed according to the method of Re et al. [36]. ABTS (PubChem CID 5815211) and Trolox (±-6-hydroxy-2,5,7,8-tetramethylchromane-2-carboxylic acid) (PubChem SID 24854347) reagents were provided by Sigma Aldrich. First, 1 mL of K_2_S_2_O_8_ water solution (2.45 mM) was added to the ABTS water solution (7 mM), mixed, and then left to react for 16 h at room temperature. The absorbance at 734 nm of the ABTS + K_2_S_2_O_8_ solution was adjusted to 0.7 using water. For sample analysis, 15 µL of sample extract and 985 µL of ABTS + K_2_S_2_O_8_ solution were mixed and incubated in darkness for 6 min. Sample absorbance was measured at 734 nm. A calibration line (R^2^ = 0.9972) in concentrations from 5 to 500 µg TE/mL was used for quantification. Results were expressed as mg Trolox Equivalents (TE) per 100 g candy.

### 2.8. 2,2-. Diphenyl-1 Picrylhydrazyl (DPPH^•^) Radical-Scavenging Activity

The DPPH assay was carried out according to the method of Brand-Williams et al. [37]. DPPH reagent (PubChem SID 57654141) was provided by Sigma-Aldrich. A solution containing 0.0035 g of DPPH in 10 mL of methanol was prepared and kept in darkness for 30 min. The absorbance at 517 nm of the DPPH solution was adjusted to 1.0 using methanol. For sample analysis, 15 µL of sample extract and 985 µL of DPPH solution were mixed and incubated in darkness for 10 min. Sample absorbance was measured at 517 nm. A calibration line (R^2^ = 0.9994) in concentrations from 10 to 500 µg TE/mL was used for quantification. Results were expressed as mg TE/100 g candy.

### 2.9. Physical Assessment

The pH was determined by dissolving 1 g of sample in 10 mL of water (50 °C) using a pH meter Crison model 2001 (Barcelona, Spain) equipped with a combined electrode, Cat. No. 52–22 (Ingold Electrodes, Wilmington, DE, USA). The moisture content (% *w*/*w*) was determined after dehydration using a D6450 drying oven (Heraeus, Boadilla del Monte, Madrid, Spain) and a BP 110S (0.001 g precision) scale (Sartorius, Alcobendas, Madrid, Spain) [38]. Instrumental colour was measured on the candy surface by reflectance using a CR-200/08 Chroma Meter II (Minolta Ltd., Milton Keynes, United Kingdom) with a D65 illumination standard, 2° observer angle, and aperture size of 50 mm. The results were expressed as CIE units: lightness (L*), redness (a*), and yellowness (b*). A Texture Profile Analysis was performed using a QTS-25 Texture Analyser (Brookfield Engineering, Harlow, Essex, England). The testing conditions were: 24 °C; TA3/100 flat cylindrical probe (20 mm in diameter); trigger point, 0.05 N; compression objective, 5 mm; cross-head speed, 0.1 mm/s; and charge cell, 10 kg. The texture variables analysed were: (i) hardness (N), the maximum force required to compress the material in the first bit; (ii) cohesiveness (no units), the ratio of the areas (force × time) resulting from the second and first bites; (iii) springiness (mm), sample height recovered during the time elapsed from the end of the first bite to the beginning of the second; and (iv) chewiness (N·mm), (hardness × cohesiveness × springiness). Adhesiveness was not included since the jelly candies were lubricated with carnauba wax. All physical measurements were made at least in triplicate.

### 2.10. Sensory Triangle Test with Consumers

In a preliminary trial, propolis resinous off-flavour was correctly identified by a trained panel when they assessed different working water solutions prepared with citric acid, sweeteners, strawberry flavour, and 0.02% PEE *w/w* (see the detailed methodology in a previous study) [3]. Thus, flavour alteration was identified as a possible sensory risk for using PEE in jelly candies. A sensory triangle test [39] was conducted to verify if propolis off-flavour was noticed or not by consumers at the doses of PEE used in jelly candies. The panel was composed of 240 consumers (145 women and 95 men, aged between 18 and 63 years old), who claimed they consumed jelly candies ranging from several times a month to several times a year. Consumers received no training. Each consumer analysed two randomly coded samples in each session.

## 3. Results

### 3.1. Quantitative Polyphenol Profile of PEE and Jelly Candies

**Figure 1:**
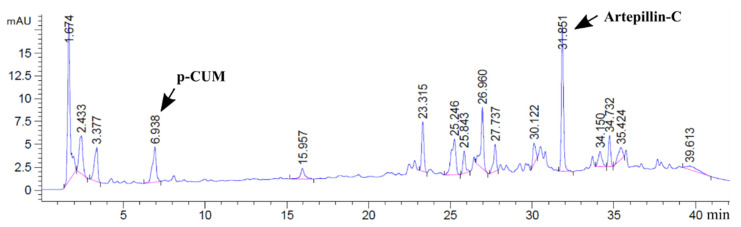
Representative chromatogram of the propolis polyphenols measured at λ = 280 nm by UV-vis and HPLC-DAD in a jelly candy sample.

A representative chromatogram of the propolis polyphenols present in a candy sample is shown in Figure 1. A total of 15 polyphenols were determined in PEE (Table 2). The most abundant compounds (% *w*/*w*) were, in decreasing order: 3,5-diprenyl-4-hydroxycinnamic acid (3.41), p-coumaric acid (1.54), 3-prenyl-4-hydroxycinnamic acid (1.52), and 2,2-dimethyl-8-prenyl-2H-1-benzopiran-6-propenoic acid (0.81). Overall, polyphenols quantified using caffeic and P-CUM acids (excluding 2,2-dimethyl-2H-1-benzopyran-6-propenoic acid and Kaempferide) accounted for 8.7% *w*/*w* of PEE. According to this quantification, the addition of PEE (P1/P2) provided 3.41/6.82 µg of DHCA and 1.54/3.08 µg of P-CUM acid per g raw candy. The contents of DHCA and P-CUM acids determined in the candies are shown in Table 3. Both phenolic acids had similar (*p >* 0.05) concentrations in the S- and F-candies. The addition of PEE increased (*p <* 0.05) the concentrations of DHCA and P-CUM acids in the P1 and P2 candies made with sugars or fructans; the average candy concentrations (µg/g) of DHCA were around 6.6 (P2 candies) and 3.3 (P1 candies), while the average candy concentrations of P-CUM were around 3.3 (P2 candies) and 1.7 (P1 candies). Thus, both propolis bioactive compounds remained almost without degradation in the final product.

### 3.2. Effects of Propolis on the Antioxidant Capacity of Jelly Candies

The antioxidant assessment of the candies is shown in Table 4. TPCs (mg GAE/100 g) were similar (*p* > 0.05) in the S- and F-candies, with strawberry candies having the highest (*p* < 0.001) values. In general, the addition of PEE increased (*p* < 0.05) TPC in the P1 and P2 candies made with sugars or fructans. The average TPCs (mg GAE/100 g) ranged from 25.1–27.1 (P2 candies), 18.8–20.0 (P1 candies), and 16.5–15.4 (untreated candies). ABTS values (mg TE/100 g) were higher (*p* < 0.05) in the S- than in the F-candies, with strawberry candies also having the highest average values (*p* < 0.001). The addition of PEE also increased (*p* < 0.05) the ABTS average values in the P1 and P2 candies made with sugars or fructans. The ABTS average values ranged from 10.2–15.3 (P2 candies), 8.0–8.3 (P1 candies), and 2.7–3.3 (untreated candies). Unlike ABTS, DPPH values (mg TE/100 g) were similar (*p* > 0.05) in the S- and C-candies, with strawberry candies having the highest values (*p* < 0.001) again. The addition of PEE (*p* < 0.05) increased DPPH values in the P1 and P2 candies made with sugars or fructans. The DPPH average values ranged from 9.4–11.3 (P2 candies), 7.0–7.3 (P1 candies), and 5.5–5.6 (untreated candies). Candy concentrations of DHCA/P-CUM correlated (*p* < 0.001) with the values of TPC (R = 0.73), ABTS (R = 0.80), and DPPH (R = 0.55), which confirmed that the presence of micro quantities of propolis polyphenols clearly improved the antioxidant capacity of jelly candies made with sugars or fructans.

### 3.3. Effects of Propolis on the Physical Properties of Jelly Candies

Physical assessment of jelly candies is shown in Table 5. The F-candies contained more (*p* < 0.001) water (around 5% *w*/*w*) than the S-candies. The moisture content was lower (*p* < 0.05) in the untreated S-candies than in the P1 and P2 S-candies, while similar (*p* > 0.05) in the untreated, P1, and P2 F-candies. The average moisture contents (% *w*/*w*) ranged from 23.9–25.0 (F-candies) and 18.3–20.0 (S-candies). The pH values were slightly higher (*p* < 0.001) in the S- than in the F-candies. The pH values were similar (*p* > 0.05) in the untreated, P1, and P2 candies. The average pH values measured were 3.4 (S-candies) and 3.2 (F-candies). Colour coordinates varied with the dye used. Blue samples (menthe) had low values of a* (positives or negatives) associated with higher negative values of b*, while both red (strawberry) and orange (orange) samples had positive chromatic values, predominating a* or b*, respectively. When samples of the same colour were compared, the S- and F-candies had similar (*p* > 0.05) CIELab values. The addition of PEE, a dark brown resin, did not affect (*p* > 0.05) the L* value in S-candies, while it increased (*p* < 0.05) the L* value in the F-candies. There were also some changes (*p* < 0.05) in the a* and *b values as a result of PEE use in the S- and F-samples. Regarding texture, the S-candies had higher (*p* < 0.001) hardness and chewiness and lower (*p* < 0.001) springiness and cohesiveness than the S-candies. The addition of PEE did not affect (*p* > 0.05) any texture attribute in S-candies, although some differences were found (*p* < 0.05) in the hardness and chewiness between P1 and P2 F-candies. Nevertheless, using PEE had no dose-dependent effect on moisture, pH, CIELab colour, or instrumental texture. L* and hardness correlated (*p* < 0.001) with moisture content (R = −0.57 and R = −0.58, respectively).

### 3.4. Identification of Propolis Off-Flavour in Jelly Candies

The results of the triangle sensory test made with consumers are shown in Table 6. In general, the number of correct identifications, in decreasing order, were: 57 (untreated vs. P2 S-candies), 44 (untreated vs. P2 F-candies), 43 (untreated vs. P1 F-candies), 41 (untreated vs. P2 S-candies), 38 (P1 vs. P2 S-candies), and 26 (P1 vs. P2 F-candies). According to the standard [39], at least 48 (*n =* 120) correct identifications are required for flavour differences between treatments to be considered significant (*p <* 0.05). On balance, there were a significant number (*p <* 0.05) of correct identifications when untreated vs. P2 S-candies were compared, while consumers did not identify (*p >* 0.05) other untreated, P1, or P2 samples.

## 4. Discussion

Polyphenol content determined in PEE by HPLC UV-vis and DAD was coherent with the supplier‘s declaration (9.9% *w*/*w*). The quantitative profile obtained, with a predominance of prenylated p-coumaric acids, caffeoylquinic acids, and diterpenic acids, agrees with those reported for other similar PEE from *Braccharis dracunculifolia*, where Artepillin-C was the most abundant polyphenol (4.04% *w*/*w*) [20]. P-CUM and Artepillin-C are considered as the main chemical markers of Brazilian green propolis [15]. Artepillin-C, 3,5-diprenyl-4-hydroxycinnamic acid, is responsible for gastroprotective, anti-inflammatory, antioxidant, antimicrobial, and antitumor propolis effects, although propolis also contains caffeic acid derivatives and other minor polyphenols with antioxidant and antimicrobial potential [40].

At the doses used in candies, PEE dissolved in the hot liquor under stirring without using solvents, as required in other trials [30]. The quantities of DHCA and P-CUM incorporated into the raw product were practically recovered in the final product, so that thermal damage was irrelevant. An unpublished study performed by differential scanning calorimetry on Brazilian green propolis found that wax melts between 60 and 70 °C, while propolis chemical components degraded in the range of 100–200 °C [41]. Therefore, it is expected that introducing PEE along with the thermo-sensitive ingredients (flavours, dyes, and acids) to the hot liquor at mild temperature (80 °C) facilitates the homogenization of dry extract and the retention of propolis polyphenols.

Using 0.2 g of PEE per kg raw product improved candy AC, regardless of the ingredients and cooking procedure used. The TPC assay measures the total reducing capacity, since the Folin–Ciocalteu reagent, a mixture of phosphomolybdate and phosphotungstate, is not specific for phenolic antioxidants. Gallic acid is used as standard to quantify antioxidant activity. The ABTS assay measures the ability of antioxidants to extinguish the ABTS• radical cation in lipophilic and hydrophilic environments. An ABTS value can be assigned to all compounds capable of scavenging the ABTS• by comparing their scavenging capacity with that of Trolox (a water-soluble vitamin E analogue). The DPPH assay monitors the chemical reactions involving the DPPH• radical, which is a scavenger for other radicals. Rate reduction of a chemical reaction upon addition of DPPH reagent is used as an indicator of the radical nature of that reaction. Trolox may also be used as standard. As seen, TPC values (mg GAE/100 g), DPPH (mg TE/100 g) and, particularly, ABTS (mg TE/100 g), increased as the concentration (µg/g) of propolis polyphenols increased in jelly candies. Therefore, these three assays can be used for assessing the changes in the antioxidant status of jelly candies as a result of PEE addition.

The antioxidant effects of propolis polyphenols were affected by the fruity dyes and flavours used in jelly candies. Strawberry candies had the highest AC compared to menthe and orange ones. The three commercial dyes used in jelly candies are based on molecules with potential antioxidants. Carminic acid [3,5,6,8-tetrahydroxy-1-methyl-9,10-dioxo-3-[(2S,3R,4R,5S,6R)-3,4,5-trihydroxy-6-(hydroxymethyl)oxan-2-yl]-9,10-dihydroanthracene-2-carboxylic acid] and curcumin [(1E,6E)-1,7-bis (4-hydroxy- 3-methoxyphenyl) -1,6- heptadiene-3,5-dione)] are two well-known phenolic compounds [42], while Idacol (Brilliant Blue FCF), a synthetic dye obtained by the condensation of 2-formylbenzenesulfonic acid and the appropriate aniline, followed by oxidation, contains aromatic rings with scavenging activity [43]. Commercial fruity flavours are prepared with a large number of chemical compounds in different proportions, including aromatic esters and other compounds that may present some reducing activities. Nature-identical flavouring agents are more resistant to candy processing conditions than nature essential oils. Either for their antioxidant properties or their stability, it is expected that different combinations of dyes and flavours may provide different antioxidant properties to jelly candies. On the other hand, carbohydrates, the major ingredients of candies, did not play a relevant antioxidant role, despite there being some compounds that may present reducing activities. Chicory inulin exhibits the best OH-scavenging activity among soluble carbohydrates, as it is a long-chain soluble polymer with many places where the reaction with·OH may derive in a proton and an electron to form a water molecule [44]. Similarly, stevia, a product containing ent-kaurene diterpenoid glycosides [45], may also have some reducing activity. Whether the candies contained sugars and starch or fructans, their AC improved by adding micro quantities of propolis antioxidants.

Propolis has been tested to date as a macro ingredient for functional candies. Rivero et al. [4,30] developed several functional jelly candies based on dry PEE (7–8% *w*/*w*). Some antioxidant, sensory, and physical properties were assessed, although the antioxidant effects of PEE on candies were not established. Dry PEE had to be previously treated with solvents and dewaxed, perhaps due to its low water solubility and the high dose used. In a first study, honey jelly candies with propolis had a higher AC (0.82 mmol TE/100 g) than several commercial ones (0.24 mmol TE/100 g), confirming the antioxidant potential of propolis in this product. In a second study, jelly candies were based on PEE combined with orange or raspberry derivatives (powder, juice, or essence). This formulation allowed an increase of the TPC (up to 551 mg GAE/100 g) and the AC (up to 1.82 mmol TE/100 g) in jelly candies. As seen, the above TPC largely exceeds those found for P2 candies in the present study, since candy formulation, including PEE dose (7% vs. 0.026% *w*/*w*), was very different. An overview of other recent trials indicates that AC may largely vary in jelly candies enriched with different fruit derivatives or plant extracts [5,7,8,9,10,12,13,14,46,47]. In these trials, phenolic ingredients were tested as nutraceutical ingredients in candies obtained under different experimental conditions (ingredients, doses, manufacturing procedures, AC assays, etc.), which makes it difficult to elucidate what ingredients present better antioxidant properties.

Candy enrichment with micro quantities of phenolic antioxidants aims to obtain a more stable product with a better nutritional value. As can be seen, the presence of propolis polyphenols at low concentrations was sufficient to enhance the antioxidant capacity of jelly candies, probably perhaps due to their good stability and radical scavenging activity. AC values of 159 mg GAE/100 g (TPC), 2.6 mg TE/100 g (ABTS), and 14.8 mg TE/100 g (DPPH) were reported in jelly candies largely based on pomegranate juice (62% *w*/*w*), a natural source of flavonoids and vitamin C [5]. These ABTS and DPPH values were not particularly high compared to those values obtained in the present study for the P2 samples, as pomegranate juice is probably a thermo-sensitive ingredient with a low dry extract. Data on fruity jelly candies enriched with micro quantities of phenolic extracts are scarce. In a previous trial [2], a similar strawberry F-candy was enriched (0.026%) with two rosemary dry extracts containing 7.4% or 14.6% polyphenols, with rosmarinic acid, a well-known natural preservative, as the main bioactive compound. Due to the addition of rosemary extract, the TPC increased from 19.0–19.7 to 28.3–41.1 mg GAE/100 g (49–108% relative increases), while AC increased from 1.4–1.5 to 3.0–5.1 mM TE (114–240% relative increases). In the present study, the addition of a lower quantity of PEE (0.02%) led to equivalent relative increases in the TPC (50%) and ABTS (190%) values of strawberry F-candies. This is evidence that propolis and rosemary polyphenols provide similar antioxidant properties when reaching similar concentrations in jelly candies.

The most relevant physical differences seen among treatments concerned candy moisture. During candy manufacturing, the hot liquor was adjusted to 82 ± 0.1 °C before moulding in an attempt to obtain products with a homogeneous moisture content. After drying, the F-candies retained more water than the S-candies, so that fructans were more hygroscopic substances than cooked sugars and gelatinized starch. PEE favoured water retention in some candies, perhaps as this extract contains some dissolved components with water binding properties. However, moisture differences among samples with and without PEE were too marked (>1% *w*/*w*) in relation to the doses added, which points to experimental drying conditions (air renovation cycle, product load, and location in the chamber, etc.) being possibly varied in some product batches. As expected, the presence of micro quantities of PEE did not produce any dose-dependent effect on colour, pH, or texture, which strongly depends on the acidifying, dying, and gelling agents used. Jelly candy was prepared with lactic and citric acid, using sodium citrate as buffer. Gelatine is responsible for gel elasticity, starch increases gel strength, while inulin is a thickening agent that provides a creamy consistency. The addition of phenolic antioxidants may potentially interfere with the gelling properties of gelatine, although higher doses than those used in the present study would be required [48]. As seen, the addition of PEE had no clear effects on any texture attribute in both, S-candies, a firmer product with less water, as F-candies, a softer product with more water. A relevant fact was that lightness and hardness decreased as moisture content increased, which may help to understand some of the results obtained. Water retention favours jelly candies being more translucid, decreasing light reflectance, and hinders the crystallization of dissolved carbohydrates, resulting in a softer product. Thus, the PEE effects observed on candy colour and texture might respond to the moisture differences that exist among candies. In other jelly candies, high doses of extracts or fruit derivatives were required to produce changes in CIELab colour or instrumental texture [4,5].

Using propolis led to some implications in candy flavour. The PEE used is a natural product that was not deodorized, showing an intense resinous off-flavour, described as bitter, pungent, and astringent. A previous approach found that a 0.02% *w*/*w* PEE might be noticed by candy consumers. In the further sensory test, consumers had certain difficulties in identifying candies with propolis off-flavour. Panellists identified propolis off-flavour in some samples when the PEE dose was increased from 0.01 to 0.02% *w*/*w*. Propolis off-flavour was more difficult to detect when jelly candy contained fructan fibres and stevia, probably because these ingredients contributed to its masking. FOS and chicory inulin have poor sweetening properties but contain fructose residues and may enhance fruity flavours in products, such as jelly candies [1]. In addition, *Stevia rebaudiana*, a sweetener with an intense bitter aftertaste, would hamper the detection of propolis bitterness. Propolis has been seen to exhibit certain sensory limitations in sugary food products. For example, a study found that honey mixed with PEE at concentrations higher than 0.5% *w*/*w* is not acceptable to consumers due to its unpleasant sensory characteristics [31], while, in another study, honey jelly candies made with propolis (7.5% *w*/*w*) were well accepted by consumers [30]. It is likely that consumers accept that a nutraceutical product based on propolis may present some off-flavour; otherwise, adverse sensory properties of propolis can be reduced with different treatments (e.g., deodorization, dewaxing, and encapsulation). However, in widely consumed jelly candies, fruity traits should predominate over other sensory traits. This is possible because the unpleasant flavour of propolis can be masked by enhancing fruit flavours, which represents a technological advantage.

## 5. Conclusions

Green propolis ethanolic dry extract is rich in phenolic antioxidants (>8.7%), with Artepillin-C as the most abundant compound (3.4%). Its incorporation into the hot liquor at mild temperature allows a good retention of propolis polyphenols in jelly candies. Very high recovery percentages (89.0–97.4% and 83.0–90.4% for P1 and P2 candies, respectively) were reached for Artepillin-C in the final product. Jelly candies made with sugars or dietetic fructans have poor antioxidant properties, which strongly depend on the fruity dyes and flavours used. Hence, their antioxidant capacity can be improved (relative increases of up to 465%) by adding micro quantities of propolis antioxidants. The required dose of propolis extract is too low to produce any relevant physical change (water retention, colour, pH, or texture) related to variations in the oxidation status of jelly candies, although propolis off-flavour may appear. Propolis, owing to its good antioxidant properties, has potential for use as a functional ingredient in healthier candy products destined for the regular consumers of candies, in particular, children and young people. Specific studies will be needed to elucidate the potential benefits of propolis for both jelly candies (shelf life and oxidative stability) and human health (bioaccessibility, bioavailability, or antioxidant status).

## Figures and Tables

**Table 1:** Jelly candy raw ingredients (% *w*/*w*).

Ingredients	S-Candies	F-Candies	All Candies
Sucrose	21		
Glucose syrup 79 °B	38.33		
Acidified thinned corn starch	10		
Fructooligosaccharides syrup 72 °B		70.58	
Inulin		11.44	
Water *	23.72	10.89	
*Stevia rebaudiana*		14	
Pork gelatine type “A”			4.1
Citric acid			0.9
Lactic acid			0.6
Sodium citrate			0.6
Flavour (menthe, orange, or strawberry)			0.3
Blue dye (Idacol) **			0.0175
Orange dye (curcumin + carminic acid at 13:3 *w*/*w*) **			0.0259
Red dye (carminic acid) **			0.0375
Propolis ethanolic dry extract (PEE)			0, 0.01 or 0.02

Abbreviations: S: Sugary; F: Fructan. * Includes the water used in ingredient solutions. ** Alternatively used for each fruity candy.

**Table 2:** Identification (HPLC-MS) and quantification (HPLC-DAD and UV-Vis) of the polyphenols present in propolis ethanolic dry extract.

Compounds	Formula	Theoretical *m*/*z*	Experimental *m*/*z*	Error	(% *w*/*w*)
Monocaffeoylquinic acid ^(1)^	C_16_ H_18_ O_9_	353.0878	353.0878	0.02	0.08
Caffeic acid	C_9_ H_8_ O_4_	179.0350	179.0355	2.89	0.11
p-Coumaric acid (P-CUM)	C_9_ H_8_ O_3_	163.0401	163.0406	3.27	1.54
3,5-di-*O* – Caffeoylquinic acid ^(1)^	C_25_ H_24_ O_12_	515.1195	515.1202	1.36	0.17
3,4-di-*O* – Caffeoylquinic acid ^(1)^	C_25_ H_24_ O_12_	515.1195	515.1206	2.14	0.02
Methyl-3,4-di-*O* – Caffeoylquinic acid ^(1),^	C_26_ H_26_ O_12_	529.1351	529.1350	0.28	0.30
Methyl-4,5-di-*O* – Caffeoylquinic acid ^(1)^	C_26_ H_26_ O_12_	529.1351	529.1346	1.04	0.09
3-Prenyl-4-hydroxy cinnamic acid ^(2)^	C_14_ H_16_ O_3_	231.1027	231.1035	3.60	1.52
2,2-dimethyl-2H-1-benzopyran-6-propenoic acid	C_14_ H_14_ O_3_	229.0870	229.0871	0.36	
4-Hydroxy-3(E)-(4-hydroxy-3-methyl-2-butenyl)-5-prenyl cinnamic acid ^(2)^	C_19_ H_24_ O_4_	315.1602	315.1610	2.59	0.19
Kaempferide	C_16_ H_12_ O_6_	299.0561	299.0569	2.64	
3-Prenyl-4-(2methylproprionyloxy)- cinnamic acid ^(2)^	C_18_ H_22_ O_4_	301.1445	301.1449	1.22	0.15
3-Hydroxy-2,2-dimethyl-8-prenyl-2H-1-benzopyran-6-propenoic acid ^(2)^	C_19_ H_24_ O_4_	315.1602	315.1610	2.59	0.01
3-Prenyl-4dihydrocinnamoyloxycinnamic acid ^(2)^	C_23_ H_24_ O_4_	363.1602	363.1607	1.42	0.02
(E)-3-{-4-hydroxy-3-[(E)-4(2,3-dihydrocinnamoyl oxy)-3methyl-2-butenyl]-5-prenylphenyl}-2-propenoic acid ^(2)^	C_28_ H_32_ O_5_	447.2177	447.2181	0.90	0.08
3,5-Diprenyl-4-hydroxy cinnamic acid ^(2)^ (DHCA)	C_19_ H_24_ O_3_	299.1653	299.1658	1.78	3.41
2,2-Dimethyl-8-prenyl-2H-1-benzopiran-6-propenoic acid ^(2)^	C_19_ H_24_ O_3_	299.1653	299.1662	3.11	0.81
3-Prenyl-4-dihydrocinnamoyloxy cinnamic acid ^(2)^	C_23_ H_24_ O_4_	363.1602	363.1607	1.42	0.21
Total polyphenols					8.71

Abbreviations: ^(^^1)^ Quantified using the calibration curve of caffeic acid, same UV spectrum. ^(2)^ Quantified using the calibration curve of p-coumaric acid, same UV spectrum [35].

**Table 3:** Remaining contents (μg/g) of p-coumaric (P-CUM) and 3,5-diprenyl-4-hydroxycinnamic (DHCA) acids in jelly candies.

				S-Candies					F-Candies		
				Menthe	Orange	Strawberry	Average	Menthe	Orange	Strawberry	Average
	PEE	Added		M	M	M	M	M	M	M	M
P-CUM	Untreated			<LoQ	<LoQ	<LoQ	<LoQ	<LoQ	<LoQ	<LoQ	<LoQ
μg/g	P1	1.54		1.61 ^b^	1.54 ^b^	2.04 ^b^	1.73 ^b^	1.79 ^b^	1.63 ^b^	1.66 ^b^	1.69 ^b^
	P2	3.08		3.26 ^a^	3.31 ^a^	3.56 ^a^	3.38 ^a^	3.32 ^a^	3.21 ^a^	2.95 ^a^	3.16 ^a^
			SEM	0.422	0.428	0.449	0.238	0.429	0.414	0.383	0.222
DHCA	Untreated			<LoQ	<LoQ	<LoQ	<LoQ	<LoQ	<LoQ	<LoQ	<LoQ
μg/g	P1	3.41		3.20 ^b^	2.95 ^b^	3.84 ^b^	3.33 ^b^	3.21 ^b^	3.02 ^b^	3.49 ^b^	3.24 ^b^
	P2	6.82		6.45 ^a^	6.93 ^a^	6.54 ^a^	6.64 ^a^	6.80 ^a^	6.49 ^a^	6.44 ^a^	6.58 ^a^
			SEM	1.237	0.898	0.849	0.467	0.882	0.823	0.835	0.462

Abbreviations: S: Sugary; F: Fructan; PEE: Propolis Ethanolic Extract; P1 and P2: 0.01% and 0.02% *w*/*w* PEE; M: Mean; SEM: Standard Error of Mean; LoQ: Limit of quantification (0.1 μg/g). ^a,b^ effects of PEE addition (*p* < 0.05) on jelly candies made with the same ingredients. Sample size: *n =* 18 per each treatment and level.

**Table 4:** Effects of PEE addition on the antioxidant capacity of jelly candies.

			S-Candies					F-Candies	
			Menthe	Orange	Average	Menthe	Orange	Strawberry	Average
	PEE		M	M	M	M	M	M	M
TPC	Untreated		12.44 ^b^	11.92 ^c^	15.36 ^c^	13.18 ^c^	14.60 ^c^	21.67 ^b^	16.48 ^c^
mg GAE/100 g	P1		13.48 ^b^	17.51 ^b^	17.81 ^b^	21.27 ^b^	17.89 ^b^	20.83 ^b^	20.00 ^b^
	P2		23.44 ^a^	22.53 ^a^	25.11 ^a^	27.93 ^a^	21.11 ^a^	32.60 ^a^	27.12 ^a^
		SEM	1.658	1.374	0.964	1.938	0.858	1.711	0.991
ABTS	Untreated		1.24 ^c^	2.20 ^c^	3.29 ^c^	2.05 ^c^	1.14 ^c^	4.98 ^c^	2.73 ^c^
mg TE/100 g	P1		5.43 ^b^	7.25 ^b^	8.29 ^b^	4.68 ^b^	7.10 ^b^	12.35 ^b^	8.04 ^b^
	P2		12.15 ^a^	16.69 ^a^	15.30 ^a^	6.90 ^a^	8.36 ^a^	15.33 ^a^	10.20 ^a^
		SEM	1.421	1.899	0.945	0.627	0.996	1.377	0.742
DPPH	Untreated		4.25 ^b^	3.88 ^b^	5.57 ^c^	4.67 ^c^	2.58 ^c^	9.49 ^c^	5.58 ^c^
mg TE/100 g	P1		4.67 ^b^	4.22 ^b^	7.05 ^b^	5.41 ^b^	5.46 ^b^	11.02 ^b^	7.29 ^b^
	P2		7.73 ^a^	10.79 ^a^	11.32 ^a^	6.30 ^a^	7.50 ^a^	14.42 ^a^	9.40 ^a^
		SEM	0.491	1.006	0.299	0.212	0.640	0.652	0.560

Abbreviations: S: Sugary; F: Fructan; PEE: Propolis Ethanolic Extract; P1 and P2: 0.01% and 0.02% *w*/*w* PEE; M: Mean; SEM: Standard Error of Mean; TPC: Total Phenolic Content; GAE: Gallic Acid Equivalent; ABTS: 2.2’-azinobis-(3-ethylbenzothiazoline-6-sulfonic); DPPH: 2,2-diphenyl-1-picrylhydrazyl radical; TE: Trolox Equivalents. ^a,b,c^ effects of PEE addition (*p* < 0.05) on jelly candies made with the same ingredients. Sample size: *n* = 18 per each treatment and level.

**Table 5:** Effects of PEE addition on the physical properties (moisture, pH, colour, and texture) of jelly candies.

			S-Candies					F-Candies		
			Menthe	Orange	Strawberry	Average	Menthe	Orange	Strawberry	Average
	PEE		M	M	M	M	M	M	M	M
Moisture	Untreated		18.86 ^b^	17.18 ^b^	17.64 ^b^	18.28 ^b^	22.90	25.24	22.64 ^b^	23.60
% *w*/*w*	P1		19.97 ^a^	20.31 ^a^	19.67 ^a^	19.98 ^a^	22.83	25.04	25.37 ^a^	24.41
	P2		19.45 ^a^	20.16 ^a^	19.31 ^a^	19.64 ^a^	23.58	24.71	26.84 ^a^	25.04
		SEM	0.142	0.527	0.316	0.137	0.686	0.187	0.686	0.289
pH	Untreated		3.38	3.48	3.36	3.41	3.19	3.17 ^a^	3.23 ^b^	3.20
	P1		3.36	3.49	3.33	3.39	3.20	3.08 ^b^	3.27 ^a,b^	3.19
	P2		3.34	3.50	3.33	3.39	3.23	3.06 ^b^	3.31 ^a^	3.20
		SEM	0.009	0.005	0.009	0.014	0.008	0.021	0.014	0.016
Lightness	Untreated		17.38	50.63	25.33	31.11	26.21 ^b^	46.40 ^b^	23.86 ^b^	32.16 ^b^
CIE units	P1		17.80	50.09	24.12	30.67	28.25 ^a^	48.51 ^a^	28.10 ^a^	34.96 ^a^
	P2		18.24	50.25	25.03	31.17	27.46 ^a^	47.89 ^a^	29.48 ^a^	34.95 ^a^
		SEM	0.203	0.107	0.355	2.838	0.350	0.335	0.861	1.909
a*	Untreated		4.71	7.95 ^b^	30.64 ^b^	14.43	−4.20	11.60	32.64	13.35
CIE units	P1		4.28	7.52 ^a^	29.06 ^a^	13.62	−6.79	11.39	33.02	12.54
	P2		4.40	9.01 ^a^	34.88 ^a^	16.10	−6.60	11.69	33.31	12.80
		SEM	0.562	0.227	0.388	2.368	0.425	0.254	0.261	3.121
b*	Untreated		−17.61	31.08	4.37 ^a^	5.95	−21.63 ^a^	27.38 ^a,b^	4.59	3.45
CIE units	P1		−18.20	31.77	2.96 ^b^	5.51	−25.28 ^b^	25.67 ^b^	3.97	1.45
	P2		−17.78	32.64	5.69 ^a^	6.85	−22.83 ^a^	28.20 ^a^	3.19	2.85
		SEM	0.192	0.876	0.402	3.992	0.568	0.451	0.226	4.041
Hardness	Untreated		5.89 ^a^	3.54	4.73 ^a^	4.72	4.71	2.71 ^a^	3.13 ^a,b^	3.38 ^a,b^
N	P1		4.56 ^b^	3.23	3.76 ^b^	4.58	4.13	2.33 ^b^	2.41 ^b^	2.90 ^b^
	P2		4.80 ^a,b^	3.80	4.24 ^a,b^	4.68	4.43	2.51 ^a,b^	4.19 ^a^	3.86 ^a^
		SEM	0.236	0.100	0.143	0.132	0.160	0.129	0.248	0.119
Springiness	Untreated		5.52	5.54	5.52	5.36	5.36	5.73	5.58	5.62
mm	P1		5.56	5.54	5.42	5.51	5.51	5.57	5.53	5.60
	P2		5.51	5.52	5.28	5.42	5.42	5.63	5.53	5.84
		SEM	0.020	0.020	0.062	0.029	0.030	0.029	0.022	0.015
Cohesiveness	Untreated		0.88	0.93	0.79	0.83	0.88	0.97	0.94	0.96
No units	P1		0.90	0.92	0.90	0.89	0.90	0.94	0.92	0.94
	P2		0.86	0.90	0.83	0.84	0.87	0.99	0.88	0.93
		SEM	0.009	0.009	0.014	0.007	0.012	0.010	0.016	0.008
Chewiness	Untreated		28.68	18.05	18.57	20.32	22.35	15.00 ^a,b^	16.30 ^a,b^	18.07 ^a^
N x mm	P1		22.76	16.43	18.43	22.30	20.50	12.17 ^b^	12.27 ^b^	15.14 ^b^
	P2		22.53	18.80	18.40	20.7	22.35	14.00 ^a,b^	19.10 ^a,b^	19.49 ^a^
		SEM	1.177	0.503	0.785	0.625	0.730	0.714	1.028	0.528

Abbreviations: S: Sugary; F: Fructan; PEE: Propolis Ethanolic Extract; P1 and P2: 0.01% and 0.02% *w*/*w* PEE; M: Mean; SEM: Standard Error of Mean. ^a,b^ effects of PEE addition (*p* < 0.05) on jelly candies made with the same ingredients. Sample size: *n* = 18 per each treatment and level.

**Table 6:** Identification of jelly candy flavour in a triangle test made by consumers.

		S-Candies		F-Candies	
	Consumer Trials	Correct Identifications	*p*-Value	Correct Identifications	*p*-Value
Menthe					
Untreated vs. P1	40	14	NS	12	NS
Untreated vs. P2	40	19	*	12	NS
P1 vs. P2	40	9	NS	7	NS
Orange					
Untreated vs. P1	40	10	NS	17	NS
Untreated vs. P2	40	17	NS	20	*
P1 vs. P2	40	13	NS	10	NS
Strawberry					
Untreated vs. P1	40	14	NS	14	NS
Untreated vs. P2	40	14	NS	12	NS
P1 vs. P2	40	13	NS	9	NS
Average					
Untreated vs. P1	120	38	NS	43	NS
Untreated vs. P2	120	50	*	44	NS
P1 vs. P2	120	35	NS	26	NS

Abbreviations: S: Sugary; F: Fructan; PEE: Propolis Ethanolic Extract; P1 and P2: 0.01% and 0.02% *w*/*w* PEE; *p*-value: probability. Levels of significance: * *p <* 0.05; and NS *p >* 0.05 (correct identifications according the ISO 4120:2004).

## Data Availability

Not applicable.

## References

[1] Delgado P., Bañón S. (2018). Effects of replacing starch by inulin on the physicochemical, texture and sensory characteristics of gummy jellies. CyTA—J. Food.

[2] Cedeño-Pinos C., Martínez-Tomé M., Murcia M.A., Jordán M.J., Bañón S. (2020). Assessment of Rosemary (*Rosmarinus officinalis* L.) Extract as Antioxidant in Jelly Candies Made with Fructan Fibres and Stevia. Antioxidants.

[3] Yan B., Davachi S.M., Ravanfar R., Dadmohammadi Y., Deisenroth T.W., Van Pho T., Odorisio P.A., Darji R.H., Abbaspourrad A. (2020). Improvement of vitamin C stability in vitamin gummies by encapsulation in casein gel. Food Hydrocoll..

[4] Rivero R., Archaina D., Sosa N., Schebor C. (2021). Development and characterization of two gelatin candies with alternative sweeteners and fruit bioactive compounds. LWT.

[5] Cano-Lamadrid M., Calín-Sánchez Á., Clemente-Villalba J., Hernández F., Carbonell-Barrachina Á.A., Sendra E., Wojdyło A. (2020). Quality parameters and consumer acceptance of jelly candies based on pomegranate juice “mollar de elche”. Foods.

[6] da Silva L.B., Annetta F.E., Alves A.B., Queiroz M.B., Fadini A.L., da Silva M.G., Efraim P. (2016). Effect of differently processed açai (Euterpe oleracea Mart.) on the retention of phenolics and anthocyanins in chewy candies. Int. J. Food Sci. Technol..

[7] Yenrina R., Sayuti K., Putri R.A. (2015). Antioxidant activity and bioactivity (LC50) of soursop leaves jelly candy with addition of soursop fruit extract (*Annona muricata* L.). Pak. J. Nutr..

[8] Vergara L.P., Reissig G.N., Franzon R.C., Carvalho I.R., Zambiazi R.C., Rodrigues R.S., Chim J.F. (2020). Stability of bioactive compounds in conventional and low-calorie sweet chewable candies prepared with red and yellow strawberry guava pulps. Int. Food Res. J..

[9] Mazur L., Gubsky S., Dorohovych A., Labazov M. (2018). Antioxidant properties of candy caramel with plant extracts. Ukr. Food J..

[10] Mandura A., Šeremet D., Ščetar M., Vojvodić Cebin A., Belščak-Cvitanović A., Komes D. (2020). Physico-chemical, bioactive, and sensory assessment of white tea-based candies during 4-months storage. J. Food Process. Preserv..

[11] Kim I., Yang M., Cho K.K., Goo Y.M., Kim T.W., Park J.H., Cho J.H., Jo C., Lee M., Lee O.H. (2013). Effect of medicinal plant extracts on the physicochemical properties and sensory characteristics of gelatin jelly. J. Food Process. Preserv..

[12] Rodríguez-Zevallos A., Hayayumi-Valdivia M., Siche R. (2018). Optimización de polifenoles y aceptabilidad de caramelos de goma con extracto de jengibre (*Zingiber officinale* R.) y miel con diseño de mezclas. Braz. J. Food Technol..

[13] Sukandar D., Radiastuti N., Muawanah A., Hudaya A. (2011). Antioxidant Activity From Water Extract Of Kecombrang Flower (*Etlingera elatior*) Leading To Jelly Candy Formulation. J. Kim. Val..

[14] Charoen R., Savedboworn W., Phuditcharnchnakun S., Khuntaweetap T. (2015). Development of Antioxidant Gummy Jelly Candy Supplemented with Psidium guajava Leaf Extract. KMUTNB Int. J. Appl. Sci. Technol..

[15] Moise A.R., Bobiş O. (2020). Baccharis dracunculifolia and dalbergia ecastophyllum, main plant sources for bioactive properties in green and red brazilian propolis. Plants.

[16] Pasupuleti V.R., Sammugam L., Ramesh N., Gan S.H. (2017). Honey, Propolis, and Royal Jelly: A Comprehensive Review of Their Biological Actions and Health Benefits. Oxid. Med. Cell. Longev..

[17] Fan Y., Yi J., Hua X., Zhang Y., Yang R. (2017). Preparation and characterization of gellan gum microspheres containing a cold-adapted β-galactosidase from Rahnella sp. R3. Carbohydr. Polym..

[18] Ccana-Ccapatinta G.V., Mejía J.A.A., Tanimoto M.H., Groppo M., de Carvalho J.C.A.S., Bastos J.K. (2020). *Dalbergia ecastaphyllum* (L.) Taub. and *Symphonia globulifera* L.f.: The Botanical Sources of Isoflavonoids and Benzophenones in Brazilian Red Propolis. Molecules.

[19] Berretta A.A., Silveira M.A.D., Cóndor Capcha J.M., De Jong D. (2020). Propolis and its potential against SARS-CoV-2 infection mechanisms and COVID-19 disease: Running title: Propolis against SARS-CoV-2 infection and COVID-19. Biomed. Pharmacother..

[20] Wang K., Hu L., Jin X.L., Ma Q.X., Marcucci M.C., Netto A.A.L., Sawaya A.C.H.F., Huang S., Ren W.K., Conlon M.A. (2015). Polyphenol-rich propolis extracts from China and Brazil exert anti-inflammatory effects by modulating ubiquitination of TRAF6 during the activation of NF-κB. J. Funct. Foods.

[21] Directive. (EC), No 46/2002. Of the European Parliament and of the Council, of 10 June 2002, on the Approximation of the Laws of the Member States Relating to Food Supplements. https://eur-lex.erupa.eu/legal-content/EN/TXT/PDF/?uri=CELEX:32002L0046.

[22] Brasilia I.N.N. (2001). Ministério da Agricultura e do Abastecimento, Secretaria de Defesa agropecuária. https://www.defesa.agricultura.sp.gov.br/legislacoes/instrucao-normativa-n-30-de-26-de-junho-de-2001,1039.html.

[23] Burdock G.A. (1998). Review of the biological properties and toxicity of bee propolis (propolis). Food Chem. Toxicol..

[24] Berretta A.A., Arruda C., Galeti F., Baptista N., Piacezzi A., Marquele-Oliveira F., Issa J., da Silva H., Damasco B., Ramos C., Shiomi V.W.N. (2017). Functional properties of Brazilian propolis: From chemical composition until the market. Superfood and Functional Food—Na Overview of Their Processing and Utilization.

[25] Galeotti F., Maccari F., Fachini A., Volpi N. (2018). Chemical Composition and Antioxidant Activity of Propolis Prepared in Different Forms and in Different Solvents Useful for Finished Products. Foods.

[26] Regulation. (CE), N°1924/2006. Scientific Opinion on the Substantiation of Health Claims Related to Propolis (ID 1242, 1245, 1246, 1247, 1248, 3184) and Flavonoids in Propolis (ID 1244, 1644, 1645, 3526, 3527, 3798, 3799). https://www.efsa.europa.eu/es/efsajuornal/pub/181c.

[27] Seibert J.B., Bautista-Silva J.P., Amparo T.R., Petit A., Pervier P., dos Santos Almeida J.C., Azevedo M.C., Silveira B.M., Brandão G.C., de Souza G.H.B. (2019). Development of propolis nanoemulsion with antioxidant and antimicrobial activity for use as a potential natural preservative. Food Chem..

[28] Spinelli S., Conte A., Lecce L., Incoronato A.L., Del Nobile M.A. (2015). Microencapsulated Propolis to Enhance the Antioxidant Properties of Fresh Fish Burgers. J. Food Process Eng..

[29] Ezazi A., Javadi A., Jafarizadeh-Malmiri H., Mirzaei H. (2021). Development of a chitosan-propolis extract edible coating formulation based on physico-chemical attributes of hens’ eggs: Optimization and characteristics edible coating of egg using chitosan and propolis. Food Biosci..

[30] Rivero R., Archaina D., Sosa N., Leiva G., Baldi Coronel B., Schebor C. (2020). Development of healthy gummy jellies containing honey and propolis. J. Sci. Food Agric..

[31] Osés S.M., Pascual-Maté A., Fernández-Muiño M.A., López-Díaz T.M., Sancho M.T. (2016). Bioactive properties of honey with propolis. Food Chem..

[32] Delgado P., Bañón S. (2015). Determining the minimum drying time of gummy confections based on their mechanical properties. CyTA—J. Food.

[33] Veiga R.S., Mendonça S., Mendes P.B., Paulino N., Mimica M.J., Lagareiro Netto A.A. (2017). Artepillin C and phenolic compounds responsible for antimicrobial and antioxidant activity of green propolis and Baccharis dracunculifolia DC. J. Appl. Microbiol..

[34] Dias L.G., Pereira A.P., Estevinho L.M. (2012). Comparative study of different Portuguese samples of propolis: Pollinic, sensorial, physicochemical, microbiological characterization and antibacterial activity. Food Chem. Toxicol..

[35] Marcucci M.C., Sawaya A.C.H.F., Custodio A.R., Paulino N., Eberlin M.N., Basic N.O.I. (2008). HPLC and ESI-MS typification: New approaches for natural therapy with Brazilian propolis. Scientific Evidence of the Use of Propolis in Ethnomedicine.

[36] Re R., Pellegrini N., Proteggente A., Pannala A., Yang M., Rice-Evans C. (1999). Antioxidant activity applying an improved ABTS radical cation decolorization assay. Free Radic. Biol. Med..

[37] Brand-Williams W., Cuvelier M., Berset C. (1995). Use of free radical method to evaluate antioxidant activity. LWT-Food Sci. Technol..

[38] Association of Official Agricultural Chemists (2000). Official methods of analysis of AOAC International.

[39] (2004). International Standards Organization-ISO Sensory Analysis-Describes a Procedure for Determining Whether a Perceptible Sensory Difference or Similarity Exists between Samples of Two Products.ISO 4120. Geneva, Switzerland: The International Organization for Standardization. https://www.iso.org./standard/33495.html.

[40] Pereira Beserra F., Gushiken L.F.S., Hussni M.F., Ribeiro V.P., Bonamin F., Jackson C.J., Pellizzon C.H., Bastos J.K. (2020). Artepillin C as an outstanding phenolic compound of Brazilian green propolis for disease treatment: A review on pharmacological aspects. Phyther. Res..

[41] Marcucci M.C., Cunha I.B.S., Sanchez E.M.S., Passarelli-Gonçalves C., Cedeño-Pinos C., Bañón S. (2021). Thermal Analysis of Brazilian propolis. Characteristics of Crude Resin, Ethanolic Extracts and Wax Isolated Compounds. Z Naturforsch C Biosci..

[42] Dikshit R., Tallapragada P. (2018). Comparative study of natural and artificial flavoring agents and dyes.

[43] Bassam M.E.A., Bassam E.A., Ali M.F. (2005). Handbook of Industrial Chemistry: Organic Chemicals.

[44] Peshev D., Vergauwen R., Moglia A., Hideg É., Van Den Ende W. (2013). Towards understanding vacuolar antioxidant mechanisms: A role for fructans?. J. Exp. Bot..

[45] Wölwer-Rieck U. (2012). The leaves of *Stevia rebaudiana* (Bertoni), their constituents and the analyses thereof: A review. J. Agric. Food Chem..

[46] da Silva L.B., Queiroz M.B., Fadini A.L., Fonseca R.C.C., Germer S.P.M., Efraim P. (2016). Chewy candy as a model system to study the influence of polyols and fruit pulp (açai) on texture and sensorial properties. LWT-Food Sci. Technol..

[47] Kim H., Cadwallader K.R., Kido H., Watanabe Y. (2013). Effect of addition of commercial rosemary extracts on potent odorants in cooked beef. Meat Sci..

[48] Wu J., Chiu S.C., Pearce E.M., Kwei T.K. (2001). Effects of phenolic compounds on gelation behavior of gelatin gels. J. Polym. Sci. Part A Polym. Chem..

